# Osteosarcoma in a ceRNET perspective

**DOI:** 10.1186/s12929-024-01049-y

**Published:** 2024-06-05

**Authors:** Nicola Mosca, Nicola Alessio, Alessandra Di Paola, Maria Maddalena Marrapodi, Umberto Galderisi, Aniello Russo, Francesca Rossi, Nicoletta Potenza

**Affiliations:** 1 Department of Environmental, Biological and Pharmaceutical Sciences and Technologies, University of Campania “Luigi Vanvitelli”, Caserta, Italy; 2 Department of Experimental Medicine, University of Campania “Luigi Vanvitelli”, Naples, Italy; 3 Department of Woman, Child and General and Specialist Surgery, University of Campania “Luigi Vanvitelli”, Naples, Italy

**Keywords:** Osteosarcoma, Non-coding RNA, ceRNA, ceRNET, Tumor microenvironment

## Abstract

Osteosarcoma (OS) is the most prevalent and fatal type of bone tumor. It is characterized by great heterogeneity of genomic aberrations, mutated genes, and cell types contribution, making therapy and patients management particularly challenging. A unifying picture of molecular mechanisms underlying the disease could help to transform those challenges into opportunities.

This review deeply explores the occurrence in OS of large-scale RNA regulatory networks, denominated “competing endogenous RNA network” (ceRNET), wherein different RNA biotypes, such as long non-coding RNAs, circular RNAs and mRNAs can functionally interact each other by competitively binding to shared microRNAs. Here, we discuss how the unbalancing of any network component can derail the entire circuit, driving OS onset and progression by impacting on cell proliferation, migration, invasion, tumor growth and metastasis, and even chemotherapeutic resistance, as distilled from many studies. Intriguingly, the aberrant expression of the networks components in OS cells can be triggered also by the surroundings, through cytokines and vesicles, with their bioactive cargo of proteins and non-coding RNAs, highlighting the relevance of tumor microenvironment. A comprehensive picture of RNA regulatory networks underlying OS could pave the way for the development of innovative RNA-targeted and RNA-based therapies and new diagnostic tools, also in the perspective of precision oncology.

## Introduction

Osteosarcoma (OS) is the most severe and common primary malignant tumor of the bone, which shows a bimodal incidence with a first peak in children, as primary bone cancer, and another peak in adults, as secondary cancer related to radiative therapies or other pathologies [1–3]. It is mainly observed in lower long bones and has a high risk of distant metastasis and invasion to the other bones and particularly to lung tissue; in fact, at the time of diagnosis, 20% of OS patients have already developed metastases, out of which 90% are lung metastases [1, 4]. Although OS represents only 5% of tumors in pediatric patients, its severity and ability to metastasize early are responsible for a high cancer-related mortality rate [1, 5]. The five-year survival rate for patients with localized OS is about 60–70%, whereas it is less than 20% for patients with metastatic OS. Chemotherapy is responsible for the impairment of bone metabolism and for the onset of osteoporosis leading to a decrease of bone mineral density in OS patients and predisposing OS long-term survivors to a high risk of bone fractures [6].

OS arises from primitive mesenchymal bone-forming cells within the osteoblastic lineage, undergoing aberrant alterations at any stage of differentiation; the bone niches and their microenvironment are strictly linked, and tumor microenvironment (TME) greatly contribute to OS progression and metastasis [1, 7–9]. Vast genomic instability and multiple genomic aberrations characterize the majority of OS cases (58%), such as gain or loss of some portions or entire chromosomes [10]. Apart from these characteristic structural alterations, large-scale sequencing analyses have also identified recurrently mutated genes, such as TP53 (lost in > 90% of OS), deletion of RB (up to 30% of OS) and other drivers lesions such as MYC amplification, PTEN loss and deletion of ATRX [10]. Related to genetic mutations, alterations in many signaling pathways, such as Notch and Wnt, contribute to osteosarcoma genesis. Most of OS cases are sporadic, however a considerable subset of cases occurs in the setting of established cancer predisposition syndromes [3, 10].

In recent years, an increasing number of studies have been published on the possible role of non-coding RNAs contributing to pathophysiology of OS, firstly regarding microRNA (2065 results retrieved by PubMed searching by keywords “osteosarcoma AND miRNA” by March 2024), then lncRNA (1024 results retrieved by PubMed searching by keywords “osteosarcoma AND lncRNA” by March 2024), and more recently circular RNA (326 results retrieved by PubMed searching by keywords “osteosarcoma AND circRNA” by March 2024).

microRNAs (miRNA) are short non-coding RNAs (approximately 20 nt long) that work by driving multiprotein complexes on complementary sequences of target transcripts, thus affecting their translation and/or stability [11]. One miRNA can bind various transcripts, and vice versa one transcript can be targeted by different miRNAs, giving rise to complex regulatory networks controlling more than 30% of protein-coding genes, thus playing key roles in almost all physiological pathways and in the pathogenesis of several diseases [12, 13]. Much evidence has shown that microRNAs can function as either oncogene by downregulating oncosuppressive proteins, or tumor suppressor by negatively regulating oncogenic targets, thus contributing to the onset and progression of osteosarcoma [14, 15]. As an example of oncomiR, miR-21 is able to down-regulate PTEN, TPM1, PDCD4, thus inducing OS cell growth, migration, invasion and metastasis [16, 17]. miR-34a is an example of oncosuppressive miRNAs, whose expression restoration could rescue the abnormal cellular processes in preclinical OS models [18]; several miR-34a oncotargets have been validated and a special link with TP53 has been also highlighted, since miR-34 is a direct transcriptional target of p53, with different feedback regulatory loops contributing to OS [19].

Long ncRNAs (lncRNAs), with a size longer than 200nt and up to several kilobases (up to 100 kb), represent the largest class of ncRNAs in the mammalian genome, and further classified into subclasses, depending on their genomic locations, origins, and transcription directions [20]. LncRNAs are structurally and functionally very versatile, so that they can interact with DNA, other RNA molecules, and proteins, thus regulating gene expression at epigenetic, transcriptional, post-transcriptional, and translational level [21]. The number of lncRNAs involved in cancer initiation and progression is continuously growing and can also be found in curated databases such as Lnc2Cancer or the Cancer LncRNA Census [22, 23]. Some lncRNAs have long been known, such as MALAT1, acting as oncogene in different tumors, including OS; many others have been more recently annotated due to the increasing advances of high-throughput RNA sequencing technologies [24].

Circular RNAs (circRNAs) are covalently closed continuous RNA loops, originated from the primary form of transcripts, mainly mRNAs; through their interaction with DNA, other coding or non-coding RNAs, and proteins, circRNAs can control gene expression at different levels, from transcriptional to post-transcriptional level [25, 26]. The expression of many circRNAs is abnormal in different cancer types, and they have been demonstrated to play relevant roles in carcinogenesis [27].

It is becoming increasingly clear that beyond “conventional” unidirectional regulation of a specific gene expression exerted by the molecules above described (e.g., one miRNA versus one target), the different RNA biotypes can engage in intricate regulatory networks, underlying physiological homeostasis and whose derailing has pathological consequences [28–30]. In fact, even unrelated and unexpected transcripts, coding or non-coding, can be functionally linked through miRNAs if they share their binding sites; in this scenario, different RNA species can regulate each other’s by competitively binding to shared miRNAs, thus titrating their availability and preventing their inhibitory binding to the other RNA targets. The binding miRNAs sites become “the letters” of an “RNA code” by which different RNA biotypes, independently from their coding potentiality, form large-scale regulatory networks across the transcriptome, reciprocally fine-tuning their expression levels and thus governing different biological pathways. The described functional relationships are denominated “competing endogenous RNA (ceRNA) networks”, abbreviated as “ceRNET”. In a physiological state, an optimal crosstalk among the RNA molecules occurs, so that the shared pool of miRNAs is sufficient to target repression and govern different biological pathways; however, the unbalancing of any network component, such as an aberrant expression, can affect the entire regulatory circuit acting as a driving force for human diseases, including carcinogenesis.

In recent years, literature concerning osteosarcoma is becoming dominated by association with non-coding RNA biology. Many studies profile microRNAs expression in different patient cohorts, finding differentially expressed miRNAs with a diagnostic/prognostic potential; some others go deeply into the mechanisms trying to piece together the molecular events and identify the governed biological pathways contributing to osteosarcoma genesis. This review aims to give a comprehensive view of RNA regulatory networks involving lncRNA-miRNA-mRNA axes and circRNA-miRNA-mRNA axes in the ceRNET perspective. Literature on PubMed published before 31 March 2024 was screened by the following keywords: “(osteosarcoma) AND (ceRNA),” retrieving 173 results; “((osteosarcoma) AND (lncRNA)) AND (axis),” retrieving 233 results; “((osteosarcoma) AND (circRNA)) AND (axis),” retrieving 123 results. Then, we selected for this study only those articles reporting experimental validation of interaction between lncRNA or circRNA versus miRNA, and miRNA versus mRNA target by RNA immunoprecipitation (RIP) and/or luciferase and/or RNA pull-down assays; duplicate results from our screening were excluded. The ceRNETs were listed grouping together those involving lncRNAs, those involving circRNAs, in alphabetic order referred to lncRNAs or circRNAs, with the aim to put together the networks governed by the same lncRNA or circRNA; the lists were further processed by subgrouping those endowed with oncogenic power, or tumor suppressor action, or involvement in chemoresistance. The described literature processing criteria led to the results presented in Tables 1, 2 and 3, discussed in the next sections.

**Table 1:** Oncogenic and tumor suppressive ceRNETs involving lncRNAs

**Competing Endogenous RNAs**					
**lncRNA**	**mRNA**	**Shared miRNA**	**Effect**	**Role**	**References**
AC007207.2	SIRT7	miR-1306-5p	oncogenic	proliferation, metastasis	[31]
ADIRF-AS1	IRS1	miR-761	oncogenic	proliferation, apoptosis, migration, invasion, tumor growth	[32]
AFAP1-AS1	TCF4	miR-4695-5p	oncogenic	proliferation, invasion, tumor growth	[33]
AFAP1-AS1	IGF1R	miR-497	oncogenic	proliferation, apoptosis, migration, invasion, tumor growth	[34]
AP003352.1	LPAR3	miR-141-3p	oncogenic	proliferation	[35]
APTR	YAP1	miR‐132‐3p	oncogenic	proliferation, apoptosis, invasion, migration	[36]
ASMTL-AS1	ADAM9	miR-342-3p	oncogenic	proliferation, apoptosis, migration, invasion, tumor growth	[37]
AWPPH	FZD7	miR-93-3p	oncogenic	proliferation, migration, invasion	[38]
BCRT1	FGF7	miR-1303	oncogenic	proliferation, EMT	[39]
BLACAT1	SOX12	miR-608	oncogenic	proliferation, migration	[40]
BSN-AS2	SYTL2	miR-654-3p	oncogenic	proliferation, apoptosis, migration, invasion	[41]
CASC9	SOX12	miR-874-3p	oncogenic	proliferation, invasion, tumor growth	[42]
CASC15	RAB14	miR-338-3p	oncogenic	proliferation, migration, invasion, tumor growth, TME	[43]
CCAL	ANGPTL4	miR-29b	oncogenic	angiogenesis	[44]
CDKN2B-AS1	CCNG1	miR-122-5p	oncogenic	proliferation, invasion	[45]
CRNDE	MRP9	miR-136-5p	oncogenic	proliferation, migration	[46]
DANCR	ROCK1	miR-335-5p, miR-1972	oncogenic	proliferation, tumor growth, metastasis	[47]
DANCR	MSI2	miR-149	oncogenic	migration, invasion	[48]
DICER1-AS1	ATG5	miR-30b	oncogenic	proliferation, migration, invasion, autophagy, tumor growth	[49]
DLEU1	DDX5	miR-671-5p	oncogenic	proliferation, migration, invasion	[50]
DLGAP1-AS2	HK2	miR-451a	oncogenic	Proliferation, migration, invasion, tumor growth; glycolysis	[51]
DLX6-AS1	DLK1	miR-129-5p	oncogenic	stemness, tumor growth	[52]
DLX6-AS1	HOXA9	miR-641	oncogenic	proliferation, apoptosis, migration, invasion, tumor growth	[53]
DLX6-AS1	RAB10	miR-141-3p	oncogenic	proliferation, migration, invasion, tumor growth	[54]
DSCAM-AS1	USP47	miR-101-3p	oncogenic	proliferation, migration, invasion, EMT	[55]
DUXAP8	TOP2A	miR-635	oncogenic	proliferation, migration, invasion	[56]
EBLN3P	RAB10	miR-224-5p	oncogenic	proliferation, migration, invasion	[57]
ERVK13-1	KLF5	miR-873-5p	oncogenic	proliferation, migration, invasion	[58]
EWS	SOX2	miR-199a-5p	oncogenic	proliferation, apoptosis	[59]
FEZF1-AS1	NUPR1	miR-4443	oncogenic	proliferation, migration, invasion, tumor growth	[60]
FEZF1-AS1	CXCR4	miR-144	oncogenic	proliferation, apoptosis, migration	[61]
FGD5-AS1	RAB3D	miR-506-3p	oncogenic	proliferation, migrasion	[62]
FGD5-AS1	G3BP2	miR-124-3p	oncogenic	proliferation, invasion	[63]
GAS6-AS2	BCAT1	miR-934	oncogenic	proliferation, apoptosis, migration, invasion	[64]
GSEC	EIF5A2	miR-588	oncogenic	proliferation, apoptosis, migration	[65]
HCG11	MMP13	miR-579	oncogenic	proliferation	[66]
HCG11	PKP2	miR-1245b-5p	oncogenic	proliferation, invasion	[67]
HCG18	FOXC1	miR-188-5p	oncogenic	proliferation, migration, invasion	[68]
HCG18	PGK1	miR-365a-3p	oncogenic	proliferation	[69]
HCP5	LOXL2	miR-29b-3p	oncogenic	proliferation, migration, invasion	[70]
HNF1A-AS1	HMGB1	miR-32-5p	oncogenic	proliferation, apoptosis, migration, invasion	[71]
HIF1A-AS2	SIRT6	miR-33b-5p	oncogenic	proliferation, apoptosis, migration, invasion, tumor growth	[72]
HOTAIR	ZEB1	miR-217	oncogenic	proliferation, apoptosis, migration, invasion	[73]
HOTAIR	SYK	miR-6888-3p	oncogenic	proliferation, migration	[74]
HOTAIRM1	RHEB	miR-664b-3p	oncogenic	proliferation, apoptosis	[75]
HOXA-AS3	TEAD1	miR-1286	oncogenic	migration, invasion, tumor growth	[76]
HOXA11-AS	ROCK1	miR-124-3p	oncogenic	proliferation, migration	[77]
HULC	HMGB1	miR-372-3p	oncogenic	proliferation, migration	[78]
ILF3-AS1	SOX5	miR-212	oncogenic	proliferation, apoptosis, migration, invasion	[79]
KCNQ1OT1	CCND2	miR-4458	oncogenic	proliferation, apoptosis, migration, invasion	[80]
KCNQ1OT1	ALDOA	miR-34c-5p	oncogenic	proliferation, apoptosis, tumor growth	[81]
KCNQ1OT1	KLF12	miR-154-3p	oncogenic	proliferation, migration, invasion, tumor growth	[82]
KCNQ1OT1	KLF7	miR-3666	oncogenic	proliferation, migration, invasion, tumor growth	[83]
KLF3-AS1	MEF2C	miR-338-3p	oncogenic	proliferation, migration	[84]
LAMTOR5-AS1	TP63	miR-23a-3p	oncogenic	pyroptosis	[85]
LBX2-AS1	BRD4	miR-597-3p	oncogenic	proliferation, migration	[86]
LEXIS	CTNNB1	miR-199a	oncogenic	proliferation, apoptosis, migration, invasion	[87]
LIFR-AS1	NFIA	miR-29a	oncogenic	proliferation, apoptosis, migration, invasion, TME	[88]
LINC00152	CDK14	miR-1182	oncogenic	proliferation, migration, invasion	[89]
LINC00265	SAT1, VAV3	miR-382-5p	oncogenic	proliferation, migration	[90]
LINC00265	USP22	miR-485-5p	oncogenic	proliferation, migration	[91]
LINC00313	FOSL2	miR-342-3p	oncogenic	proliferation, migration	[92]
LINC00319	NFIB	miR-455-3p	oncogenic	proliferation, migration	[93]
LINC00460	FADS1	miR-1224-5p	oncogenic	proliferation, migration	[94]
LINC00467	KPNA4	miR-217	oncogenic	proliferation, apoptosis, migration, invasion	[95]
LINC00511	MAEL	miR-618	oncogenic	proliferation, apoptosis, migration, invasion	[96]
LINC00511	E2F1	miR-185-3p	oncogenic	proliferation, invasion	[97]
LINC00588	TP53	miR-1972	oncogenic	proliferation, apoptosis, migration, invasion	[98]
LINC00612	SOX4	miR-214-5p	oncogenic	proliferation, migration	[99]
LINC00662	NOTCH2	miR-15a-5p	oncogenic	proliferation, migration, invasion, tumor growth	[100]
LINC00662	SIK2	miR-103a-3p	oncogenic	proliferation, migration, invasion	[101]
LINC00662	ITPR1	miR-16-5p	oncogenic	proliferation, migration, invasion, stemness	[102]
LINC00662	ELK1	miR-30b-3p	oncogenic	proliferation, migration, invasion	[103]
LINC00665	WNT2B	miR-1249-5p	oncogenic	proliferation, apoptosis, migration, invasion, EMT	[104]
LINC00689	SOX18	miR-655	oncogenic	proliferation, apoptosis, migration, invasion	[105]
LINC00691	PTEN	miR-9-5p	oncogenic	proliferation, migration	[106]
LINC00707	AHSA1	miR-3383p	oncogenic	proliferation, migration, invasion	[107]
LINC00881	MMP2	miR-29c-3p	oncogenic	metastasis	[108]
LINC00909	HOXD9	miR-875-5p	oncogenic	proliferation, EMT, tumor growth	[109]
LINC00958	CEMIP	miR-4306	oncogenic	proliferation, apoptosis, migration, invasion	[110]
LINC00963	FN1	miR-204-3p	oncogenic	proliferation, migration	[111]
LINC01123	GLI1	miR-516b-5p	oncogenic	proliferation, migration	[112]
LINC01128	MMP2	miR-299-3p	oncogenic	proliferation, migration, invasion	[113]
LINC01140	HOXA9	miR-139-5p	oncogenic	proliferation, migration	[114]
LINC01278	PTHR1	miR-133a-3p	oncogenic	proliferation, migration	[115]
LINC01278	KRAS	miR-134-5p	oncogenic	proliferation, migration	[116]
LINC01410	NDRG3	miR-122-5p	oncogenic	proliferation, migration	[117]
LINC01410	HMGA2	miR-497-5p	oncogenic	proliferation, migration	[118]
LINC01419	PDRG1	miR-519a-3p	oncogenic	proliferation, EMT, tumor growth	[119]
LINC01535	KCNC4	miR-214-3p	oncogenic	proliferation, migration	[120]
LINC01614	SNX3	miR-520a-3p	oncogenic	proliferation, invasion	[121]
LINC02381	CDCA4	miR-503-5p	oncogenic	proliferation, migration	[122]
MALAT1	TGFA	miR-376a	oncogenic	proliferation	[123]
MALAT1	HMGB1	miR-142–3p/ miR-129–5p	oncogenic	proliferation, apoptosis	[124]
MALAT1	ROCK1, ROCK2	miR-144-3p	oncogenic	proliferation, migration, invasion, tumor growth and metastasis	[125]
MALAT1	HDAC4	miR-140-5p	oncogenic	proliferation, apoptosis, tumor growth	[126]
MALAT1	CDK9	miR‐206	oncogenic	proliferation	[127]
MALAT1	Rac1	miR-509	oncogenic	proliferation	[128]
MALAT1	c-Met, SOX4	miR-34a/c-5p, miR-449a/b	oncogenic	proliferation, migration	[129]
MALAT1	CCND1	miR-34a	oncogenic	proliferation, migration, invasion, tumor growth	[130]
MALAT1	NRSN2	miR-143	oncogenic	proliferation, migration, tumor growth, TME	[131]
MALAT1	ROCK1	miR-873-5p	oncogenic	proliferation, migration	[132]
MIAT	VEGFC	miR-128-3p	oncogenic	proliferation, apoptosis, migration, invasion	[133]
MIAT	SIX1	miR-141-3p	oncogenic	proliferation, migration	[134]
MIR205HG	TWIST2	miR-2114-3p	oncogenic	invasion, metastasis	[135]
MRPL23-AS1	MYH9	miR-30b	oncogenic	proliferation, invasion, tumor growth	[136]
MRUL	FUT4	miR-125a-5p	oncogenic	proliferation, migration	[137]
MSC-AS1	CDK6	miR-142	oncogenic	proliferation, apoptosis, migration, invasion	[138]
MYOSLID	RAB13	miR-1286	oncogenic	proliferation, apoptosis, migration, invasion	[139]
NBR2	FKBP11	miR-129-5p	oncogenic	metastasis	[140]
NEAT1	HIF‐1α	miR‐186‐5p	oncogenic	proliferation, invasion, EMT, tumor growth	[141]
NEAT1	TGF‐β1	miR‐339‐5p	oncogenic	proliferation, apoptosi, migration, invasion	[142]
NEAT1	HOXA13	miR-34a-5p	oncogenic	proliferation, apoptosis	[143]
NEAT1	STAT3	miR-483	oncogenic	migration, invasion, EMT, tumor growth, metastasis	[144]
NORAD	KLF10	miR-30c-5p	oncogenic	proliferation, migration, TME	[145]
NR2F1-AS1	BIRC5	miR-485-5p/miR-218-5p	oncogenic	proliferation, apoptosis, migration, invasion	[146]
NR2F2-AS1	HMGB2	miR-425-5p	oncogenic	proliferation, apoptosis	[147]
ODRUL	MMP2	miR-3182	oncogenic	proliferation, migration, invasion, tumor growth	[148]
OIP5-AS1	CDK14	miR-223	oncogenic	proliferation, apoptosis, tumor growth	[149]
OR3A4	G6PD	miR-1207-5p	oncogenic	proliferation, migration	[150]
PART 1	BAMBI	miR-20b-5p	oncogenic	proliferation, migration	[151]
PCAT-1	ZEB1	miR-508-3p	oncogenic	proliferation, migration	[152]
PCAT6	TGFBR1/2	miR-185-5p	oncogenic	proliferation, migration, invasion	[153]
PCAT6	ZEB1	miR-143-3p	oncogenic	proliferation, migration	[154]
PURPL	PDZD2	miR-363	oncogenic	proliferation, apoptosis, migration, invasion, TME	[155]
PVT1	HK2	miR-497	oncogenic	proliferation, invasion	[156]
RGMB-AS1	ANKRD1	miR-3614-5p	oncogenic	proliferation, invasion, apoptosis	[157]
ROR	YAP1	miR-185-3p	oncogenic	proliferation, migration	[158]
RP11-361F15.2	CPEB4	miR-30c-5	oncogenic	proliferation, migration	[159]
RUSC1-AS1	NOTCH1	miR-101-3p	oncogenic	proliferation, EMT, metastasis	[160]
SCAMP1	ZEB2	miR-26a-5p	oncogenic	proliferation, migration	[161]
SNHG1	WNT2B	miR-577	oncogenic	proliferation, migration, EMT, tumor growth	[162]
SNHG1	NOB1	miR-326	oncogenic	proliferation, tumor growth, metastasis	[163]
SNHG1	S100A6	miR-493-5p	oncogenic	proliferation, tumor growth	[164]
SNHG1	FGF2	miR-424-5p	oncogenic	proliferation, migration, invasion	[165]
SNHG3	RAB22A	miRNA-151a-3p	oncogenic	migration, invasion	[166]
SNHG5	ROCK1	miR-26a	oncogenic	proliferation, migration, invasion	[167]
SNHG10	FZD3	miR-182-5p	oncogenic	proliferation, migration and tumor growth	[168]
SNHG10	WTAP	miR-141-3p	oncogenic	proliferation, apoptosis, migration, invasion	[169]
SNHG12	NOTCH2	miR-195-5p	oncogenic	proliferation, migration, invasion	[170]
SNHG12	IGF1R	miR-195-5p	oncogenic	proliferation, migration, tumor growth, metastasis	[171]
SNHG14	FBXO22	miR-433–3p	oncogenic	proliferation, apoptosis, migration, invasion	[172]
SNHG15	TRAF4	miR-346	oncogenic	proliferation, apoptosis, invasion, tumor growth	[173]
SNHG16	BCL9	miR-1301	oncogenic	proliferation, migration, invasion	[174]
SNHG16	ITGA6	miR-488	oncogenic	migration, invasion, EMT, tumor growth, metastasis	[175]
SNHG20	RUNX2	miR-139	oncogenic	proliferation, apoptosis, invasion	[176]
SNHG25	SOX4	miR-497-5p	oncogenic	proliferation, migration, invasion, apoptosis, tumor growth	[177]
SOX21-AS1	IRS2	miR-7-5p	oncogenic	proliferation, migration, invasion	[178]
TMPO-AS1	E2F1	miR-329	oncogenic	proliferation, apoptosis, migration, invasion	[179]
TMPO-AS1	WNT7B	miR-199a-5p	oncogenic	proliferation, migration	[180]
TRPM2-AS	PPM1D	miR-15b-5p	oncogenic	proliferation, migration	[181]
TTN-AS1	TFAP4	miR-16–1-3p	oncogenic	proliferation, apoptosis, migration, invasion	[182]
TUG1	POU2F1	miR-9-5p	oncogenic	proliferation, apoptosis, tumor growth	[183]
TUG1	EZH2	miR-144-3p	oncogenic	migration, EMT	[184]
TUG1	ROCK1	miR-335-5p	oncogenic	migration, invasion	[185]
TUG1	FOXA	miR-212-3p	oncogenic	proliferation, apoptosis, tumor growth	[186]
TUG1	SOX4	miR-132-3p	oncogenic	proliferation, apoptosis	[187]
TUG1	HIF-1α	miR-143-5p	oncogenic	proliferation, angiogenesis, tumor growth, metastasis	[188]
TUG1	PFN2	miR-140-5p	oncogenic	proliferation, migration, invasion, tumor growth	[189]
TYMSOS	NETO2	miR-101-3p	oncogenic	proliferation, migration	[190]
UCA1	CREB1	miR-582	oncogenic	proliferation, apoptosis, migration, invasion	[191]
UCA1	E2F5	miR-513b-5p	oncogenic	proliferation, migration	[192]
WAC-AS1	SOX2	miR5047	oncogenic	proliferation, migration, invasion, tumor growth	[193]
XIST	RSF1	miR-193a-3p	oncogenic	proliferation, invasion	[194]
XIST	RAP2B	miR-320b	oncogenic	proliferation, invasion	[195]
XIST	SNAI1	miR-153	oncogenic	migration, invation, EMT	[196]
XIST	RAB16	miR-758	oncogenic	migration, invation, EMT	[197]
XLOC_005950	PFKM	miR-542-3p	oncogenic	proliferation, apoptosis	[198]
ZFAS1	APEX1	miR-135a	oncogenic	proliferation, apoptosis, migration, invasion	[199]
ZFAS1	NOB1	miR-646	oncogenic	proliferation, apoptosis, migration, invasion	[200]
GAS5	MYL9	miR-663a	tumor suppressor	proliferation, migration, apoptosis	[201]
GAS5	RHOB	miR-663a	tumor suppressor	proliferation, migration, invasion	[202]
LINC00261	PTEN	miR-620	tumor suppressor	increasing apatinib effectivennes	[203]
LINC00691	ST5	miR-1256	tumor suppressor	proliferation, migration, invasion, EMT, tuomor growth	[204]
LINC00891	TET1	miR-27a-3p	tumor suppressor	proliferation, migration, invasion	[204]
MEG3	FoxM1	miR‐361‐5p	tumor suppressor	proliferation, migration, invasion, EMT, apoptosis	[205]
MIR22HG	TET3	miR-629-5p	tumor suppressor	proliferation, apoptosis	[206]
NR_027471	TP53INP1	miR-8055	tumor suppressor	proliferation, migration, invasion, EMT, apoptosis, tuomor growth	[207]
NR_136400	TUSC5	miR-8081	tumor suppressor	proliferation, migration, invasion, EMT, apoptosis, tuomor growth	[208]
SNHG22	NKIRAS2	miR-4492	tumor suppressor	proliferation, migration, invasion, EMT, tuomor growth	[209]
TUSC7	RASSF6	miR-181a	tumor suppressor	proliferation, migration, invasion, apoptosis, tuomor growth	[210]
TUSC8	EHd2	miR-197-3p	tumor suppressor	proliferation, migration, invasion, EMT, apoptosis	[211]
XIST	PDCD4	miR-21-5p	tumor suppressor	proliferation, migration, invasion, apoptosis, tuomor growth	[212]

**Table 2:** Oncogenic and tumor suppressive ceRNETs involving circRNAs

**Competing Endogenous RNAs**					
**circRNA**	**mRNA**	**Shared miRNA**	**Effect**	**Role**	**References**
circ_0000004	PLOD2	miR-1303	oncogenic	migration, metastasis	[213]
circ_0000073	FADS2	miR-1184	oncogenic	lipid metabolism	[214]
circ_0000253	SP1	miR-1236-3p	oncogenic	proliferation, apoptosis	[215]
circ_0000282	XIAP	miR-192	oncogenic	proliferation, apoptosis	[216]
circ_0000285	IGFBP3	miR-409-3p	oncogenic	proliferation, migration, invasion, apoptosis, tumor growth	[217]
circ_0000285	TGFB2	miRNA-599	oncogenic	proliferation, migration	[218]
circ_0000376	HK2/LDHA	miR-577	oncogenic	proliferation, invasion, apoptosis, tumor growth, glycolysis	[219]
circ_0000376	BCL2	miR-432-5p	oncogenic	proliferation, migration, invasion, tumor growth	[220]
circ_0000527	ARL2	miR-646	oncogenic	proliferation, cell cycle, inflammatory mediators’ secretion	[221]
circ_0001721	MAPK7	miR-372-3p	oncogenic	proliferation, migration, invasion, EMT; glycolysis	[222]
circ_0001722	RUNX2	miR-204-5p	oncogenic	proliferation, invasion, tumor growth	[223]
circ_0001785	HOXB2	miR-1200	oncogenic	proliferation, apoptosis, tumor growth	[224]
circ_0002052	STX6	miR-382	oncogenic	proliferation, migration, invasion	[225]
circ_0002137	IGF1R	miR-433-3p	oncogenic	proliferation, invasion, metastasis, tumor growth	[226]
circ_0003074	KPNA4	miR-516b-5p	oncogenic	proliferation, migration, invasion, apoptosis, tumor growth	[227]
circ_0003732	CCNA2	miR-545	oncogenic	proliferation	[228]
circ_0003732	CPEB1	miR-377-3p	oncogenic	cell proliferation, migration, invasion, tumor growth	[229]
circ_0003998	KLF10	miR-197-3p	oncogenic	proliferation, invasion	[230]
circ_0005721	TEP1	miR-16-5p	oncogenic	proliferation, invasion, apoptosis	[231]
circ_0005909	HGMA1	miR-338-3p	oncogenic	proliferation	[232]
circ_0005909	HMGB1	miR-936	oncogenic	colony formation, migration, invasion, EMT, tumor growth	[233]
circ_0007031	HOXB6	miR-196a-5p	oncogenic	proliferation, migration, tumor growth, cytokine modulation, stemness	[234]
circ_0007534	SOX5	miR-219a-5p	oncogenic	proliferation, colony formation, migration, invasion, tumor growth	[235]
circ_0009910	IL6R	miR-449a	oncogenic	proliferation, cell cycle, apoptosis	[236]
circ_0010220	STX6	miR-198	oncogenic	proliferation, migration, invasion, cell cycle, apoptosis, tumor growth	[237]
circ_0010220	CDCA4	miR-503-5p	oncogenic	proliferation, migration, invasion, cell cycle, apoptosis, tumor growth	[238]
circ_001350	CNOT7	miR-578	oncogenic	proliferation, migration, invasion	[239]
circ_001422	FGF2	miR-195-5p	oncogenic	proliferation, migration, invasion, tumor growth	[240]
circ_0016347	KCNH1	miR-1225-3p	oncogenic	proliferation, migration, invasion; glycolysis	[241]
circ_0016347	caspase-1	miR-214-3p	oncogenic	proliferation, invasion, metastasis, tumor growth	[242]
circ_0020378	MAPK1	miR-556-5p	oncogenic	proliferation, migration, tumor growth	[243]
circ_0028171	IKBKB	miR-218-5p	oncogenic	proliferation, migration, invasion, tumor growth	[244]
circ_0032463	LEF1	miR-498	oncogenic	proliferation, migration, apoptosis	[245]
circ_0032463	PNN	miR-330-3p	oncogenic	proliferation, invasion, apoptosis	[246]
circ_0051079	MAFB	miR-1286	oncogenic	proliferation, migration, invasion, tumor growth	[247]
circ_0051079	TRIM66	miR-625-5p	oncogenic	proliferation, migration, invasion, angiogenesis, apoptosis	[248]
circ_0056285	TRIM44	miR-1244	oncogenic	proliferation, apoptosis, tumor growth; glycolysis	[249]
circ_0078767	CDK14	miR-330-3p	oncogenic	proliferation, migration, invasion, tumor growth	[250]
circ_0084582	JAG1	miR-485-3p	oncogenic	proliferation, migration, invasion, angiogenesis	[251]
circ_0096041	LIN28A	miR-556-5p	oncogenic	proliferation, migration, invasion, tumor growth	[252]
circ_0102049	MDM2	miR-1304-5p	oncogenic	proliferation, migration, invasion, apoptosis, tumor growth	[253]
circ_0136666	CEP55	miR-1244	oncogenic	proliferation, migration, invasion, tumor growth; glycolysis	[254]
circ_03955	MTDH	miR-3662	oncogenic	proliferation, migration, apoptosis, EMT, tumor growth	[255]
circABCC1	HDAC4	miR-591	oncogenic	proliferation, migration, invasion, autophagy, apoptosis, tumor growth	[256]
circANKIB1	PAX3	miR-217	oncogenic	proliferation, migration, invasion, apoptosis, tumor growth	[257]
circATRNL1	LDHA	miR-409-3p	oncogenic	apoptosis, tumor growth; glycolysis	[258]
circBLNK	GPX4	miR-188-3p	oncogenic	proliferation, apoptosis, tumor growth	[259]
circCCDC66	PTP1B	miR-338-3p	oncogenic	proliferation, migration, invasion	[260]
circCDK14	GAB1	miR-520a-3p	oncogenic	proliferation, migration, invasion, tumor growth	[261]
circCDK14	E2F2	miR-198	oncogenic	proliferation, migration, invasion, apoptosis, tumor growth; glycolysis	[262]
circCNST	LDHA/PDK1	miR-578	oncogenic	proliferation, migration, invasion, apoptosis, tumor growth; glycolysis	[263]
circCYP51A1	KLF12	miR-490-3p	oncogenic	proliferation, migration, invasion, glycolysis, hypoxia, tumor growth	[264]
circDOCK1	LEF1	miR-936	oncogenic	proliferation, migration, invasion, angiogenesis, tumor growth	[265]
circEMB	EGFR	miR-3184-5p	oncogenic	proliferation, migration, apoptosis, tumor growth, metastasis	[266]
circECE1	RAB3D	miR-588	oncogenic	proliferation, migration, invasion, apoptosis, tumor growth	[267]
circEPSTI1	MCL1	miR-892b	oncogenic	proliferation, migration, invasion	[268]
circFAM120B	PTBP1	miR-1205	oncogenic	proliferation, invasion, tumor growth	[269]
circFAT1(e2)	HK2	miR-181b	oncogenic	proliferation, migration	[270]
circHIPK3	HDAC4	miR-637	oncogenic	proliferation, migration, invasion	[271]
circHIPK3	STAT3	miR-637	oncogenic	proliferation, migration, invasion	[272]
circKIF4A	SLC7A11	miR-515-5p	oncogenic	proliferation, ferroptosis, in vivo metastasis	[273]
circLRP6	HDAC4 and HMGB1	miR-141-3p	oncogenic	proliferation, migration, invasion	[274]
circMGEA5	ZEB1 and Snail	miR-153‐3p, miR‐8084	oncogenic	migration, invasion, EMT, in vivo metastasis	[275]
circMMP9	CHI3L1	miR-1265	oncogenic	proliferation, migration, invasion	[276]
circMYO10	RUVBL1	miR-370-3p	oncogenic	proliferation, migration, EMT, tumor growth, in vivo metastasis	[277]
circNRIP1	AKT3	miR-532-3p	oncogenic	proliferation, migration, invasion, tumor growth	[278]
circPIP5K1A	YAP	miR-515-5p	oncogenic	proliferation, migration, invasion, apoptosis, cancer cell stemness, tumor growth	[279]
circPVT1	c-FLIP	miR-205-5p	oncogenic	proliferation, migration, invasion, EMT	[280]
circPVT1	CCNB1	miR-26b-5p	oncogenic	proliferation, migration, invasion, apoptosis, tumor growth, in vivo metastasis	[281]
circPVT1	FOXC2	miR-526b	oncogenic	migration, invasion	[282]
circPVT1	HAVCR2	miR490-5p	oncogenic	proliferation, migration, invasion	[283]
circRAB3IP	TWIST1	miR-580-3p	oncogenic	proliferation, migration, invasion, tumor growth	[284]
circRBMS3	EIF4B and YRDC	miR-424	oncogenic	proliferation, migration, invasion, tumor growth	[285]
circRNF220	Survivin	miR-330-5p	oncogenic	proliferation, migration, invasion, tumor growth	[286]
circSIPA1L1	MAP3K9	miR-379-5p	oncogenic	proliferation, invasion	[287]
circSRSF4	RAC1	miR-224	oncogenic	proliferation, migration, invasion, tumor growth	[288]
circTADA2A	CREB3	miR-203a-3p	oncogenic	proliferation, migration, invasion, tumor growth	[289]
circTNPO1	WNT5A	miR-578	oncogenic	proliferation, invasion, tumor growth	[290]
circUBAP2	HMGB2	miR-637	oncogenic	proliferation, migration, invasion, apoptosis, tumor growth	[291]
circUBAP2	YAP1	miR-641	oncogenic	proliferation, invasion, EMT	[292]
circUBAP2	HMGA2	miR-204-3p	oncogenic	proliferation, migration, invasion, apoptosis	[293]
circXPO1	XPO1	miR-23a-3p, miR-23b-3p, miR-23c, and miR-130a-5p	oncogenic	proliferation, invasion, apoptosis	[294]
circXPR1	DDX5	miR-214-5p	oncogenic	proliferation	[295]
circ_0000658	IRF2	miR-1227	tumor suppressor	proliferation, invasion, apoptosis, tumor growth, in vivo metastasis	[296]
circ_0002052	APC2	miR-1205	tumor suppressor	proliferation, migration, invasion, apoptosis, tumor growth	[297]
circ_0008259	PDCD4	miR-21-5p	tumor suppressor	proliferation, migration, invasion, apoptosis	[298]
circ_0008792	ZFP1	miR-711	tumor suppressor	proliferation, migration, invasion, apoptosis, tumor growth	[299]
circ_0046264	SFRP1	miR-940	tumor suppressor	proliferation, migration, invasion	[300]
circ_0069117	PF4V1	miR-875-3p	tumor suppressor	proliferation, migration	[301]
circ_0088212	APOA1	miR-520	tumor suppressor	proliferation, migration, invasion, tumor growth	[302]
circ_0102049	PLK2	miR-520 g-3p	tumor suppressor	proliferation, migration, invasion, apoptosis, tumor growth	[303]
circMTO1	KLF6	miR-630	oncogenic	proliferation, migration, invasion, apoptosis	[304]
circROCK1-E3/E4	PTEN	miR-532-5p	tumor suppressor	proliferation, migration, tumor growth, in vivo metastasis	[305]
circVRK1	ZNF652	miR-337-3p	tumor suppressor	proliferation, migration, invasion, tumor growth	[306]
circWWC3	PDE7B	miR-421	tumor suppressor	proliferation, migration, invasion, apoptosis, tumor growth	[307]

**Table 3:** ceRNETs contributing to drug response

Competing Endogenous RNAs		Shared miRNA	Drug resistance	References
**lncRNA**	**mRNA**			
FGD5-AS1	WNT5A	miR-154-5p	doxorubicin	[308]
GAS5	TP53INP1	miR- 26b-5p	cisplatin	[309]
HOTAIR	STAT3	miR-106a-5p	cisplatin	[310]
IGF2-AS	MSH6	miR-579-3p	cisplatin	[311]
LINC00161	IFIT2	miR-645	cisplatin	[312]
LINC00210	GFRA1	miR-342-3p	radiosensitivity	[313]
LINC00641	MCL1	miR-320d	cisplatin	[314]
LINC01116	HMGA2	miR-424-5p	doxorubicin	[315]
MEG3	AKT2	miR-200b-3p	Multi-drug	[316]
MIR17HG	SP1	miR-130a-3p	cisplatin	[317]
LUCAT1	ABCB1	miR-200c	methotrexate	[318]
OIP5-AS1	FOSL2	miR-377-3p	cisplatin	[319]
OIP5-AS1	PTN	miR-137-3p	doxorubicin	[320]
OIP5‐AS1	FN1	miR‐200b‐3p	doxorubicin	[321]
PVT1	CCND1	miR-15a-5p/miR-15b-5p	doxorubicin	[322]
ROR	ABCB1	miR-153-3p	cisplatin	[323]
SARCC	Hexokinase 2	miR-143	cisplatin	[324]
SNHG12	MCL1	miR-320a	doxorubicin	[325]
SNHG14	SLC7A11	miR-206	nutlin-3a	[326]
SNHG15	GFRA1	miR-381-3p	doxorubicin	[327]
SNHG15	ZNF32	miR-335-3p	cisplatin	[328]
SNHG16	ATG4B	miR-16	cisplatin	[329]
Sox2OT-V7	ULK1, ATG4A, ATG5/ULK1	miR-142/miR-22	doxorubicin	[330]
TTN-AS1	MBTD1	miR-134-5p	cisplatin	[331]
circ_0000006	BDNF	miR-646	doxorubicin	[332]
circ_0001258	GSTM2	miR-744-3p	multi-drug	[316]
circ_0003496	KLF12	miR-370	doxorubicin	[333]
circ_0004674	FBN1	miR-342-3p	doxorubicin	[334]
circ_0004674	MCL1	miR-142-5p	doxorubicin	[335]
circ_0010220	IL-6	miR-574-3p	doxorubicin	[336]
circ_0081001	TGM2	miR-494-3p	methotrexate	[337]
circCHI3L1.2	LPAATβ	miR-340-5p	cisplatin	[338]
circDOCK1	IGF1R	miR-339-3p	cisplatin	[339]
circEMB	GFR	miR-3184-5p,	methotrexate	[266]
circITCH	RASSF6	miR-524	doxorubicin	[340]
circPRKAR1B	FZD4	miR-361-3p	cisplatin	[341]
circPRDM2	EZH2	miR-760	doxorubicin	[342]
circPVT1	KLF8	miR-24-3p	multi-drug	[343]
circPVT1	TRIAP1	miR-137	doxorubicin	[344]
circSAMD4A	KLF8	miR-218-5p	doxorubicin	[345]

## CeRNETs involving lncRNAs

An increasing number of studies demonstrated that lncRNAs are key players in osteosarcomagenesis, triggering different molecular pathways involved in biological processes such as cell proliferation, migration, invasion, apoptosis, tumor growth and metastasis.

**Fig. 1:**
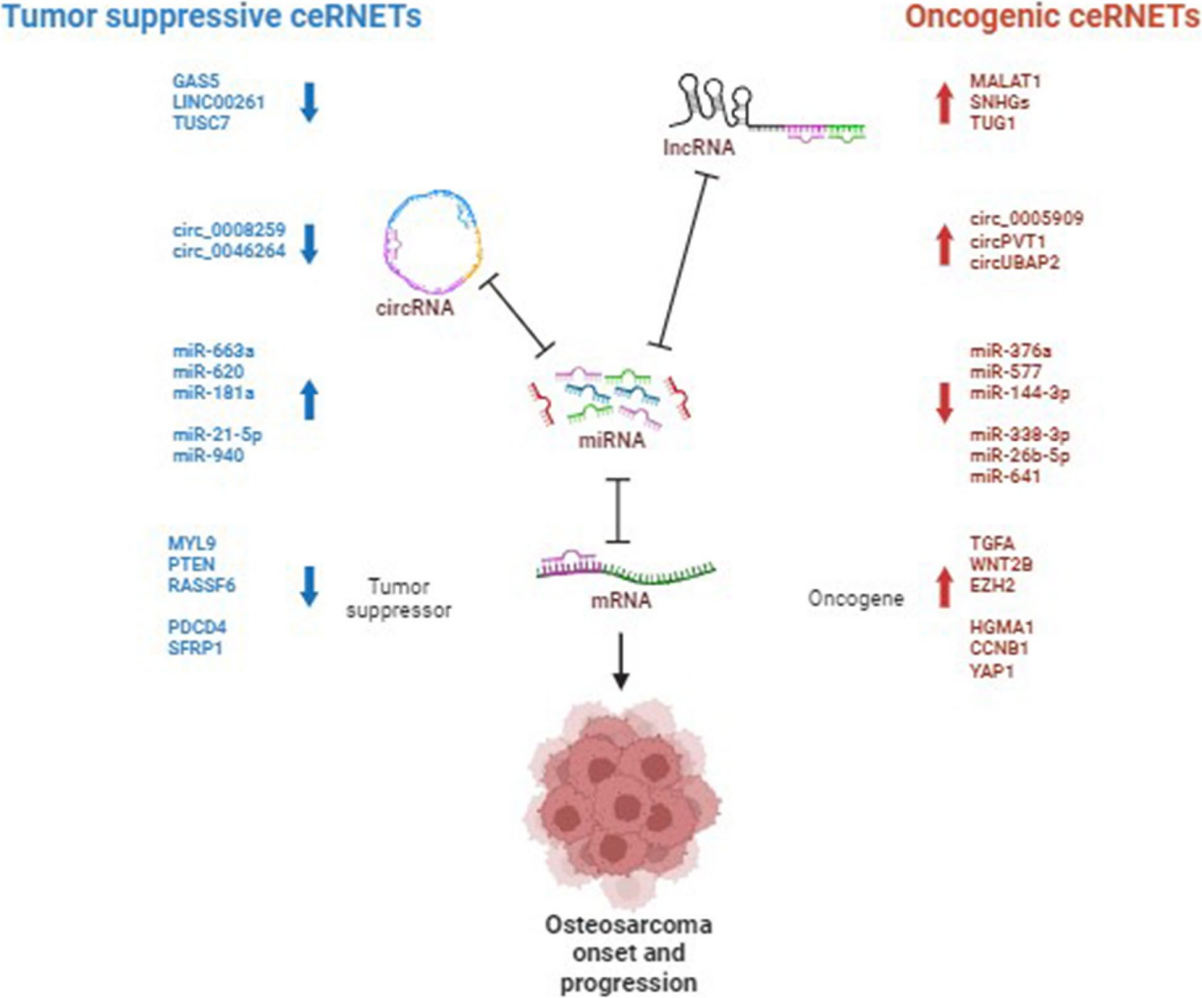
Competing endogenous RNA networks (ceRNET) relying on lncRNAs, circRNAs, miRNAs and mRNAs in osteosarcoma. Coding and non-coding RNAs can compete for binding to a shared pool of miRNAs. Optimal ceRNA crosstalk occurs in a physiological homeostasis condition; however, an aberrant expression of any circuit component can derail the network, thus contributing to the OS onset and progression by repressing tumor suppressive activities (left side) or prompting oncogenic activities (right side). The up and down arrows indicate increased or decreased expression, respectively. Some representative examples discussed in the text are reported in the figure. Figure created with BioRender.com

In this section, we discuss the role of lncRNAs as oncogenes and then as tumor suppressors through their ceRNA activity, as distilled from many studies (Fig. 1). The extensive list of lncRNAs, mechanisms and phenotypic effects is reported in Table 1.

### Oncogenic ceRNA activity

Different biological processes and molecular pathways have been demonstrated to play a key role in OS onset and progression. Among them, the early metastatic potential is a feature of OS; ceRNA activity of many lncRNAs greatly contribute to cell invasiveness and metastasis, a key to a poor prognosis (Table 1). They are also able to contribute to the “Warburg effect”, i.e. a metabolic switch from oxidative phosphorylation to aerobic glycolysis that leads to the enhancement of cell proliferation, and the rapid growth of tumor. In fact, although aerobic glycolysis is less efficient in the generation of ATP, it increases proliferation, inhibits apoptosis, and generates signaling metabolites to enhance cancer cell survival under stressful conditions, such as hypoxia. Regarding molecular pathways, different ceRNETs involving lncRNAs are consistently indicated to be able to trigger PI3K/AKT/mTOR and Wnt/beta-catenin signaling pathways, indeed promoting cell proliferation, invasion and metastasis and inhibiting apoptosis. Then, ceRNA activity can have an impact on the entire transcriptome of OS cells, when lncRNAs share miRNA binding sites with transcripts encoding transcription factors or chromatin remodeling enzymes, that can result upregulated by an overexpression of the sponging lncRNA, with pathological consequences. Among the 118 oncogenic lncRNAs reported in Table 1, below we discuss some of them consistently and more frequently reported (from 16 to 3 papers per lncRNA) to be involved in ceRNETs prompting osteosarcomagenesis, also in relation to biological processes and molecular pathways cited above.

Different members of lncRNA SNHGs family (small nucleolar RNA host genes) have received increasing attention regarding their roles in multiple bone diseases, since their unique expression profile during osteoblast differentiation and involvement in relevant pathways for osteogenesis of mesenchymal stem cells [346]. Furthermore, various lncRNAs SNHGs have been already demonstrated to be involved in different human cancers, including glioma, esophageal cancer, HCC, lung adenocarcinoma and gastric cancer [347–352]. Nine members of the family have been also reported to drive osteosarcomagenesis through different ceRNETs (Table 1). Among them, SNHG1 is consistently reported to be upregulated in OS tissues and cells, correlated with tumor size, TNM stage and lymph node metastasis, predicting poor overall survival [162–165]. In particular, the lncRNA is able to promote proliferation, migration, tumor growth and metastasis, as demonstrated in vitro and in vivo experiments through the ceRNA activity involving the miR-326/NOB1, miR-493/S100A6 and miR-577/Wnt2B axes, with the last one also activating the Wnt/beta-catenin signal pathway, one of the most critical pathway relevant for both cell proliferation and metastasis [162–164]. SNH10 is also able to activate the Wnt/beta-catenin pathway by promoting the beta-catenin transfer into nucleus to maintain the activation of the Wnt signaling by miR-182-5p/FZD3 axis, as demonstrated by in vitro and in vivo experiments [168]. The ability to promote tumor growth and metastasis in animal models by ceRNA activity has also been demonstrated for other members of the SNHGs family, i.e. SNHG12, SNHG15 and SNHG16 (Table 1). In addition, different members of the family are also involved in chemoresistance (Table 3).

MALAT1 (Metastasis associated lung adenocarcinoma transcript 1) is a well-known oncogenic lncRNA that is upregulated in several types of tumors, including lung, breast, cervical, and nasopharyngeal cancers [353]. Other studies also support its upregulation in OS tissues and cells, correlation with a poor prognosis and an oncogenic role in the initiation and progression of osteosarcoma, mainly performed by sponging specific miRNAs, as detailed below. In 2016, Luo W. et al. showed that knockdown of MALAT1 in osteosarcoma cells inhibited cell proliferation. This effect was attributed to the ability of MALAT1 to sponge miR-376a thus upregulating TGFA (Transforming Growth Factor Alpha). Since then, several reports confirmed the pro-proliferative activity of MALAT1 toward osteosarcoma cells and showed concomitant inhibition of apoptosis and induction of migration (Table 1). In particular, MALAT1 shares miR-144-3p binding sites with ROCK1 and ROCK2, two small G proteins, belonging to the Rho family, regulating cytoskeletal activities and pericellular matrix degradation involved in cell movement, proliferation and migration/invasion; indeed, the ceRNET MALAT1/miR-144-3p/ROCK1/2 is a molecular mechanism contributing to the ability of the lncRNA to promote tumor growth and lung metastasis, as demonstrated in vivo [125]. The oncogenic power of MALAT1 was also demonstrated in vivo by other ceRNA mechanisms involving the upregulation of histone deacetylase HDAC4 and the cyclin D1 via miR-140-5p and miR-34a, respectively [126, 130]. Worth of note, in 2021 Li F. et al. pointed out that MALAT1 may be released to osteosarcoma cells by surrounding cells. In particular, Bone Marrow Mesenchymal Stem Cells-Derived Extracellular Vesicles (BMSC-EVs) were found able to promote proliferation, invasion and migration of osteosarcoma cells via the MALAT1/miR-143/NRSN2/Wnt/beta-catenin axis both in vitro and in vivo, as detailed in a next section.

The lncRNA TUG1 (Taurine upregulated 1) is abnormally expressed in many cancer types and reported as an oncogene promoting cell proliferation, glycolysis, metastasis, angiogenesis and chemoradioresistance [354]. Consistently, TUG1 has been found upregulated in OS tissues and cells, and highly correlated with clinical stage, metastasis, and poor prognosis; through its ceRNA activity, it is able to increase the expression of different targets, thus promoting cell proliferation, migration, invasion, tumor growth and metastasis, as consistently demonstrated in vitro and in vivo [183–185]. TUG1 is also a relevant mediator of crosstalk between cancer-associated fibroblasts and OS cells in TME to promote invasion and distant metastasis, as detailed in a next section [188]. Different molecular pathways are triggered by TUG1 via miRNAs-sponging, such as the Wnt/beta-catenin activation and chromatin remodeling by increasing the expression of miR-144-3p target, EZH2, an H3K27me3 methyltransferase able to epigenetically silence different tumor suppressor genes [184]. In addition, TUG1 increased expression is able to turn the transcriptome of OS cells, up-regulating the transcription factors POU2F1, FOXA1, and HIF-1alfa, by sponging their targeting miRNAs, i.e. miR-9-5p, miR-212-3p, miR-143-5p, respectively, thus deeply contributing to OS progression [183, 186, 188].

KCNQ1OT1 (KCNQ1 Opposite Strand/Antisense Transcript 1) is a lncRNA transcribed in the antisense direction to the KCNQ1 gene, in the chromosomal region 11p15.5 containing two clusters of imprinted genes; KCNQ1OT1 is exclusively expressed from the paternal allele, however it is abnormally expressed from both chromosomes in most patients with the imprinting disorder of Beckwith-Wiedemann syndrome, and in multiple types of cancers (https://www.genecards.org) [355]. In particular, KCNQ1OT1 has been widely reported to be a cancer promoter in various types of tumors, such as non-small cell lung carcinoma, colorectal cancer, tongue cancer, and breast cancer [356–359]. Recently, it has been reported as a powerful oncogene also in OS, contributing to cell proliferation, migration, invasion, and tumor growth and correlating with a worse prognosis [80–83]. Its overexpression has a deep impact on the transcriptome of OS cells, since it is able to sponge miR-3666 and miR-154-3p, thus upregulating two members of Kruppel-like family of transcription factors, KLF7 and KLF12, respectively, and indeed activating the Wnt/beta‑catenin signaling [82, 83]. In addition, KCNQ1OT1 contributed to the Warburg effect by sponging miR-34c-5p and thus acting as a ceRNA for the mRNA encoding the key glycolytic enzyme aldolase A (ALDOA), thereby increasing its expression and contributing to glucose metabolism reprogramming [81].

Another lncRNA indicated as an oncogene in OS is LINC00662; it was found upregulated in OS tissues and cells, and correlated with poor prognosis; through its ceRNA activity, LINC00662 is able to promote cell proliferation, migration, invasion and tumor growth [100–103]. In particular, by miRNAs-sponging LINC00662 can enhance the expression of the mammalian Notch receptor NOTCH2 and IP receptor type 1 (ITPR1), and of a member of ETS family ELK1, thus eliciting signaling pathways reported to be involved in OS progression [100, 102, 103].

The lncRNA NEAT1 (Nuclear enriched abundant transcript 1) has been recognized as an important regulator of the expression of different genes, including some involved in cancer progression and as an activator of Wnt/beta-catenin pathway in OS, similarly to that reported for non-small-cell lung carcinoma (NSCLC) [141, 360]. It is upregulated in OS tissues and cell lines and high NEAT1 expression was associated with advanced clinical stage, distant metastasis, and poor overall survival of patients, consistent with data reported for breast cancer [141–143, 361]. In OS, its ceRNA activity greatly contribute to the promotion of cell proliferation, migration, invasion, tumor growth and metastasis; in particular, it is able to competitively bind to miR‐186‐5p, miR-339‐5p, miR-34a-5p, and miR-483, thus upregulating HIF‐1alfa, the cytokine TGF‐β1, HOXA13, and STAT3, as indicated in Table 1.

Similarly to NEAT1, the lncRNA DLX6-AS1 works as an oncogene in OS by triggering the Wnt signaling and augmenting stemness of OS cells through miR-129-5p/DLK1 axis, as demonstrated by in vitro and in vivo experiments [52]. Other studies consistently confirmed that the lncRNA is upregulated in OS tissues and cells, correlates with poor patient survival, and mechanistically contributes to OS hallmarks by the other ceRNETs reported in Table 1 [53, 54].

### Tumor suppressive ceRNA activity

A minority of lncRNAs involved in OS are reported as tumor suppressors through their ceRNA activity. One example is represented by GAS5, reported to play a tumor suppressive role in several cancers, associated with clinic-pathological traits and patient survival, and functionally involved in in cell proliferation, apoptosis, invasion, epithelial–mesenchymal transition (EMT), metastasis, and drug resistance, via multiple molecular mechanisms [362]. In OS tissues and cells GAS5 expression level was found significantly decreased in comparison to normal tissues and cells, as expected for a tumor suppressor gene. Furthermore, its overexpression suppresses OS cell proliferation, migration, and invasion in vitro; vice versa, miR-663a is highly expressed in osteosarcoma and promotes cell proliferation and migration by down-regulating its targets, MYL9 and RHOB [201, 202]. Gas5 and miR-663 are functionally linked, since Gas5 is able to sponge the miRNA: when Gas5 is down-regulated, the entire ceRNET is derailed due to the increased level of the miRNA [201, 202, 362].

Another lncRNA downregulated in OS tissues and cell lines compared with the normal ones is TUSC7. Consistently, its experimental overexpression was able to inhibit OS cell proliferation, migration and invasion, and inhibit tumor growth in vivo. Mechanistically, TUSC7 exert its role by sponging miR‑181a, resulting in an increased level of the miRNA target RASSF6 [210]. Similar results have been reported for another lncRNA, TUSC8, acting through miR-197-3p/EHD2 axis, overall pointing to possible new therapeutic approaches based on the enhancement of the tumor suppressive lncRNAs [211].

In this regard, the tumor suppressive lncRNA LINC00261 has been found to potentiate the aptanib drug effectiveness [203]. Apatinib has been recently identified as a potential treatment option for OS; its mechanism of action is well characterized, since it is a high-affinity selective inhibitor of VEGFR2; however, it is also known that different drugs can even engage ncRNAs contributing to their effectiveness [363, 364]. This is the case of apatinib, since it was demonstrated that the drug is able to increase the expression of LINC00261, that in turn can sponge miR-620, thus up-regulating the miRNA target PTEN, a well-known oncosuppressor; importantly, the activation of LINC00261/miR-620/PTEN ceRNET by Apatinib has been demonstrated also in vivo, suggesting LINC00261 as a promising target to improve the efficacy of Apatinib.

## CeRNETs involving circRNAs

CircRNAs are aberrantly expressed in almost all types of cancer [365, 366], including OS [367]. In this section, we discuss how different oncogenic circRNAs contribute to the OS onset and progression through the ceRNA mechanism, focusing the attention on those involved in cell invasiveness, Warburg effect, PI3K/AKT/mTOR and Wnt/beta-catenin signaling pathways, and chromatin remodeling, the same biological process and molecular pathways highlighted in the previous section. Then, we examine ceRNETs involving circRNAs functioning as tumor suppressors to hinder malignant growth (Fig. 1). The extensive list, their molecular mechanisms and phenotypic effect is reported in Table 2.

### Oncogenic ceRNA activity

As described above, cell invasiveness plays a pivotal role in OS progression. Interestingly, several circRNAs have been found to be involved in the modulation of OS invasiveness. For instance, Yan and colleagues found that circPVT1 is upregulated in OS tissues and is more commonly overexpressed in samples with lung metastasis. Moreover, they demonstrated that downregulation of circPVT1 can reduce cell migration and invasion via regulating of miR-526b/FOXC2 axis. Likewise, another study demonstrated that circPVT1 facilitates OS invasion and metastasis via enhancing cell epithelial–mesenchymal transition (EMT). At the molecular level, circPVT1 may enhance the invasion and metastasis by releasing c-FLIP through the interaction with miR-205-5p, highlighting a new ceRNA network [280]. Furthermore, knockdown of circPVT1 can notably inhibit the severity of tumor metastasis in lung tissues of mice modulating the 26b-5p/CCNB1 axis [281]. Consistently, circPVT1 was also found upregulated in several cancers, such as bladder cancer, oral squamous cell carcinoma, and small cell lung cancer, highlighting its relevance in cancer progression. [368–370]**.** Another circRNA involved in the regulation of metastasis in different tumor types, including OS, is circUBAP2 [371–374]. Upregulation of circUBAP2 is found in OS tissues and is associated with short survival of patients, TNM stage and distant metastasis. In addition, circUBAP2 knockdown can significantly inhibit OS cell proliferation, migration and invasion in vitro by sponging miR-204 -3p to upregulate HMGA2. Additionally, circUBAP2 can regulate cell invasion and tumor growth in vivo by regulating the miR-637/HMGB2 axis [291]. Also, CircUBAP2 knockdown increased the expression of E-cadherin while it downregulated Vimentin, two markers of EMT, thus inhibiting cell invasion. Wu and colleagues demonstrated that circUBAP2 can exert that effect through upregulating the expression of YAP1 by targeting miR‑641 in OS cells [292]. Yap1 is a key element in the Hippo signaling pathway that plays an important role in the control of cell proliferation, EMT and metastasis [375]. In this regard, the circPIP5K1A can contribute to cancer cell stemness by targeting miR‑515‑5p/YAP axis. Shi and colleagues demonstrated that circPIP5K1A knockdown, or miR-515-5p mimic, repressed the protein levels of ALDH1 and Nanog, while miR-515-5p inhibitor or YAP overexpression can reverse this effect [279]. A similar role in promoting cancer progression and metastasis has been found for other tumor types. For instance, circPIP5K1A may function as a miR-600 sponge to facilitate non-small cell lung cancer proliferation and metastasis by promoting HIF-1α [376]. Moreover, circPIP5K1A can regulate glioma progression by modulating the miR-515-5p/TCF12/PI3K/AKT axis [377]. In gastric cancer, circPIP5K1A can regulate PI3K/AKT pathway through miR-671-5p/KRT80 axis [378]. Those cross-cancer insights highlight the strong and general involvement of ncRNA function in cancer.

As discussed in the previous section, cancer cells can enhance their metabolism for rapid growth, and one of the most common metabolic changes is enhanced glycolysis, the “Warburg effect”. Several studies indicated that hyperactive glycolysis is the main metabolic alteration in OS and it is involved in cell growth, invasion, and treatment effectiveness [379]. Interestingly, circRNAs have been also described to be involved in the regulation of glucose metabolism in OS through the ceRNA mechanism, as well as lncRNAs. For instance, it has been demonstrated that circATRNL1 overexpression promoted glucose uptake and lactate production thus accelerating the Warburg effect. Mechanistically, circATRNL1 can sponge mir-409-3p to upregulate the expression level of LDHA, a key enzyme in the glycolytic pathway [258]. Likewise, Hu and colleagues demonstrated that circCNST knockdown can decrease glucose consumption, lactate production, and ATP/ADP ratio downregulating LDHA through the circCNST-miR578-LDHA/PDK1 ceRNA regulatory network [263]. Another circRNA involved in glucose metabolism is circ_0056285 which can regulate the expression of TRIM44 by sponging miR-1244, which in turn can regulate the expression of key enzymes such as HK2 and LDHA [249]. Moreover, the circCYP51A1, that was upregulated under hypoxia conditions, can markedly induce the lactate production and glucose consumption by sponging miR-490-3p and regulating KLF12. Interestingly, the knockdown of circCYP51A1 in xenograft mice models can reduce tumor growth by downregulating KLF12 and consequently reducing glycolysis associated markers, such as GLUT1, HK2 and LDHA [264].

Alteration of different molecular pathways is known to be involved in OS onset and progression. Among them, the PI3K/AKT/mTOR signaling pathway has been demonstrated to have the ability to enhance the cell cycle, inhibit apoptosis, and promote cellular proliferation, invasion, and metastasis in OS [380]. Interestingly, several studies indicate that circRNAs may play an important role in the regulation of the PI3K/AKT/mTOR pathway. In this regard, circ_001422, that is found to be upregulated in OS tissues and correlated with clinical features, can promote proliferation and metastasis, in vitro and in vivo, via the miR-195-5p/FGF2/PI3K/Akt axis [240]. Shi and colleagues demonstrated that circNRIP1 derived from BMSC-EVs can upregulate AKT3 expression by competitively binding to miR-532-3p, thus promoting proliferation and tumor growth activating the PI3K/AKT/mTOR pathway. This pathway is also triggered by circNRIP1 to promote gastric cancer progression via miR-149-5p sponging [381]. Moreover, circ_0005909 may increase viability and invasion of OS upregulating expression of HGMA1 through sponging miR-338-3p, which activated PI3K-Akt signaling pathway [232].

Another pathway playing a crucial role in OS development is the Wnt/β-catenin signaling pathway. The Wnt/β-catenin pathway is a well-known oncogenic pathway responsible for cell fate determination, stem cell replication, survival, differentiation, cell polarity, and osteogenic differentiation [380, 382]. In this regard, circ_001350, that is upregulated in OS tissues, is able to activate the Wnt pathway by inducing the β-catenin protein expression and its downstream effector cyclin D1, and c-myc. Xu and colleagues demonstrated that circ_001350 can regulate the Wnt pathway and the malignant progression by regulating the miR-578/CNOT7 axis [239]. Moreover, circMYO10 was found to regulate the Wnt signaling to induce proliferation and EMT in OS cells. At the molecular level, circMYO10 can sponge miR-370-3p and upregulate RUVBL1 expression to promote the interaction between RUVBL1 and β-catenin/LEF1 complex and thus promoting Wnt/β-catenin signaling. Interestingly, the authors demonstrated that circMYO10/miR-370-3p/RUVBL1 axis enhanced the transcription activity of β-catenin/LEF1 via histone H4K16 acetylation [277].

Histone modification is a dynamic process that alters the structure of chromatin, leading to the expression or repression of local genes. In cancer, the normal balance between active and repressive histone modification modifies the expression of oncogenes and tumor suppressor genes, leading to tumorigenesis. Recently, some evidence highlighted that deregulation of genes involved in these processes has been associated with OS tumorigenesis, progression and chemoresistance [383]. circLRP6, that is upregulated in OS and is associated with poor prognosis, was found to enhance the expression of histone deacetylase 4 (HDAC4) in OS cells via sponging miR-141-3p promoting cell proliferation, invasion [274]. The relevance of histone modification in OS was also demonstrated by Wang and colleagues showing that circABCC1 knockdown can stop the malignant progression of OS by attenuating HDAC4 expression through sponging miR-591, highlighting a new ceRNA network [256].

### Tumor suppressive ceRNA activity

Although most of the annotated circRNAs are reported to be oncogenic, different circRNAs have been found to act as tumor suppressors in OS.

For instance, circ_0046264 may exert a tumor-suppressive role in OS. Low expression of circ_0046264 was found in OS tissues and correlated with larger tumor. At cellular level, circ_0046264 can inhibit the proliferation, migration and invasion of OS cells. Du and colleagues, demonstrated that SFRP1, that is known to inhibit the proliferation, migration and invasion of OS cells by inhibiting Wnt/β-catenin signaling [384], is upregulated in OS cells overexpressing circ_0046264. Mechanistically, circ_0046264 can upregulate SFRP1 expression by sponging miR-940 [300]. The tumor-suppressive role of circ_0046264 was also demonstrated in lung cancer where it can inhibit viability, invasion, and induce apoptosis by upregulating BRCA2 expression through down-regulating miR-1245 [385].

It is well recognized that abnormal activation of the AKT/mTOR signaling pathway is one pivotal cause of OS development and progression [380]. PF4V1 is a negative regulator of the AKT signaling pathway and negatively regulates OS progression [386]. Interestingly, circ_0069117 might promote the expression of PF4V1 by sponging miR-875-3p, thus regulating the progress of OS [301].

Also, circ_0000658 was found to inhibit cell proliferation and invasion in vitro and impede tumor growth in vivo. At the molecular level, circ_0000658 can exert a tumor suppressive effect by targeting the miR-1227/IRF2 axis in OS cells [296].

Moreover, circ_0088212, which is poorly expressed in osteosarcoma tissues and cells, may function as a tumor suppressor by inhibiting cell proliferation and invasion and limiting tumorigenesis in vivo through miR-520 h/APOA1 axis [302]. Likewise, circ_0102049 could suppress the progression of OS by activating PLK2 by targeting miR-520 g-3e [303].

CircROCK1-E3/E4, a circular RNA derived from exons 3 and 4 of the ROCK1 gene, was found downregulated in OS patients with lymph node metastasis and distant metastasis. Liu and colleagues demonstrated that expression of circROCK1-E3/E4 was partially regulated by QKI, a well-known RNA Binding Protein (RBP) belonging to the STAR family of KH domain-containing RBPs. Moreover, they demonstrated that overexpression of circROCK1-E3/E4 may inhibit cell proliferation and lung metastasis in vivo by regulating miR-532-5p/PTEN axis in osteosarcoma [305].

Mir-21 is a well-known oncomiR also for OS [16, 17]. Interestingly, circ_0008259, which is downregulated in OS, can increase PDCD4 expression via adsorbing miR-21 and repressing the OS progression, thus depicting a new ceRNET involved in tumor suppressive activity [298].

## CeRNETs in chemoresistance

Osteosarcoma treatment typically involves surgery and chemotherapy; radiation therapy might be an option in certain situations. In the 1970s, amputation or limb-sparing surgery represented the standard OS treatment, yielding a 5-year survival rate of only 20%; then, chemotherapy agents elevated the post-treatment 5-year OS survival rate. The current treatment strategy usually consists of several weeks of neoadjuvant preoperative chemotherapy followed by the surgical removal of primary tumor, and also several weeks of postoperative adjuvant chemotherapy [387]. Indeed, the 5-year survival rate has increased to 70%-80% by the wide resection surgery combined with adjuvant chemotherapy. However, long-term chemotherapy poses the risk that the patient’s cells develop resistance to the chemotherapeutic drug, even to combinations of different ones, culminating in OS recurrence, distant metastasis, and treatment failure. In fact, the 5-year survival rate of patients who experience chemoresistance decreased to less than 20%. The present standard treatment chemotherapy mainly consists in the combined administration of high dose methotrexate, doxorubicin and cisplatin (MAP) [1, 387, 388]. Methotrexate is a folate analogue designed to inhibit dihydrofolate reductase; reduced folate (tetrahydrofolate) is the proximal single carbon donor in several reactions involved in the de novo synthetic pathway for purines and pyrimidines, formation of polyamines, and transmethylation of phospholipids and proteins; as consequence of methotrexate treatment, the malignant cells become starved for the purine and pyrimidine precursors of DNA and RNA and unable to synthesize DNA and RNA and proliferate [389]. Doxorubicin acts in the cancer cell according to two proposed mechanisms: by intercalating into DNA and disrupting topoisomerase-II-mediated DNA repair and by generating free radicals with consequent damage to cellular membranes, DNA and proteins [390]. Cisplatin mode of action has been linked to its ability to crosslink with the purine bases on the DNA, thus interfering with DNA repair mechanisms, causing DNA damage, and subsequently inducing apoptosis in cancer cells [391].

The effectiveness of chemotherapy in OS is markedly impacted by chemoresistance. Presently, there exist no conventional methods to overcome chemotherapy resistance in malignancies without inducing adverse side effects. The knowledge of molecular mechanisms underlying drug and multidrugs resistance is essential to investigate potential strategies for reversing this process and avoid the high doses with severe side effects [392]. Numerous studies have linked OS chemotherapy resistance to abnormal expression of different ncRNA biotypes (lncRNA, circRNA and miRNA) and it is now increasingly clear that they can mechanistically contribute to OS chemoresistance. Also considering that single mechanisms don’t fully explain chemotherapeutic resistance, but many factors can be responsible for drug resistance, the study of large-scale RNA regulatory networks can be useful to explore innovative RNA-based and RNA-targeted therapy that surmount and/or prevent chemotherapy resistance, even in a perspective of personalized treatment. Table 3 reports validated networks contributing to chemoresistance.

Most of ceRNETs involving lncRNAs contribute to cisplatin resistance, whereas most of ceRNETs involving circRNAs contribute to doxorubicin resistance; indeed, there are also some examples of ceRNETs responsible for multidrug resistance.

An example of lncRNAs enhancing cisplatin resistance is HOTAIR: in different OS cell lines it promoted the cisplatin resistance by regulating cell proliferation, invasion, and apoptosis via miR-106a-5p/STAT3 Axis [310]. HOTAIR seems to be particularly linked to cisplatin resistance, since it can induce that drug resistance also in other tumors, such as non-small cell lung cancer and nasopharyngeal carcinoma [393, 394]. OIP5-AS1 contributes to cisplatin resistance via miR-377-3p/FOSL2 axis [319], but also to doxorubicin resistance by different molecular axis, i.e. miR-137-3p/PTN [320] and miR-200b-3p/fibronect-1 axis [321]; the lncRNA is significantly upregulated in OS chemo-resistant tissues and cell lines and its knock-down reduced doxorubicin resistance in vitro and in vivo [320]. In particular, fibronectin-1, a glycoprotein related to cellular adhesion and migration processes, was demonstrated to be functionally related to the oncogenic OIP5-AS1, because the lncRNA is able to sponge the shared miR-200b-3p; this mechanism could explain fibronectin-1 upregulation in the chemo-resistant OS cell lines and tissues and its relation to unfavorable prognosis [321]. Different members of lncRNA SNHGs family can drive osteosarcomagenesis through various ceRNETs, as shown in Table 1 and discussed in a previous section; they can also contribute to chemoresistance (Table 3). As an example, SNHG15 can contribute to both cisplatin and doxorubicin resistance through miR-335-3p/ZNF32 and miR-381-3p/GFRA1 axes, respectively [327, 328]. Intriguingly, p53, very frequently lost in OS, is able to transcriptionally repress SNHG15, thus depicting regulatory pathways wherein p53 dysfunction substantially increased SNHG15 expression, that in turn sponges specific miRNAs, thus downregulating their oncogenic targets [328]. Aberrant expression of SNHG15 can also contribute to the resistance of lung adenocarcinoma and breast cancer cells to gefitinib and cisplatin, respectively, highlighting its general relevance in drug resistance [395, 396].

Whole-transcriptome sequencing of three paired multi-drug chemoresistant and chemosensitive OS cell lines and exploitation of different interaction predictive tools have highlighted how extensive and relevant such regulatory networks are, placing in functional relation unexpected lncRNAs or circRNAs with mRNAs via miRNAs [316]. Then, luciferase, RIP and RNA-pull down assays were used to validate different ceRNETs, such as that involving circ_0001258 through miR-744-3p/GSTM2 axis or another one involving circ_0004674 through miR-142-5p/MCL1 axis [316, 335]. Circ_0004674 promoted the DXR resistance also through Wnt/β-catenin pathway via regulating the miR-342-3p/FBN1 axis [334]. Among circRNAs, circPVT1 also contributed to doxorubicin resistance, as demonstrated in vitro and in vivo; it contributes to tumor growth; its silencing increased the drug sensitivity of osteosarcoma in vivo*;* it has an increased expression in DXR-resistant osteosarcoma tissues and cells. TP53-regulated inhibitor of apoptosis 1 (TRIAP1), an apoptosis inhibitor, paralleled circPVT1 increased expression in OS and in fact, they are functionally linked through miR-137, since circPVT1 is able to sponge miR-137, thus de-repressing TRIAP1 [344]. The role of PVT1 in chemoresistance was also extended to cisplatin and methotrexate other than doxorubicin by demonstrating its ability to sponge miR-24-3p and thus up-regulating KLF8 [343].

Overall, those studies turn the spotlight on intricate RNA regulatory networks underlying chemotherapeutic drug resistance mechanisms, inspiring new strategies based on management of such networks, e.g. by down-regulating or overexpressing any network component functionally linked in the ceRNET, and possibly overcome, revert and even prevent chemoresistance, prospectively also in terms of precision medicine.

## ceRNETs contributing to TME relevance

The tumor microenvironment (TME) plays a key role in OS onset and progression. TME is a mixture of cancer and non-cancer cells and their stroma, that can be categorized in two major categories of components: cellular components, including different cell types, such as osteoblasts, osteoclasts, mesenchymal stem cells, cancer-associated fibroblasts (CAFs), endothelial cells, adipocytes and immune cells, especially tumor-associated macrophages (TAMs); acellular components, such as the extracellular matrix (ECM), cytokines, growth factors and extracellular vesicles (EVs), with their bioactive cargo of proteins and different RNA biotypes [397].

Cells in the TME are in constant autocrine and paracrine communication, which contributes to tumor development, progression, drug resistance and metastasis. The local microenvironment provides a fertile niche for tumor growth, wherein interaction between cancer and bone cells leads to an increase in OS cell proliferation and altered bone remodeling. In particular, a crucial role is played by TAMs which represent the most abundant cells of TME and are involved in tumor growth and progression [398]. Macrophages exist in two different phenotypes: the classically activated macrophages M1 and the alternatively activated macrophages M2 [7]. M1 macrophages exhibit pro-inflammatory and anti-cancer effects by releasing pro-inflammatory cytokines and inducible factors against pathogens; instead, M2 ones have anti-inflammatory, pro-tumoral and pro-angiogenic properties [7]. It has been reported that the prevalence of M2 phenotype in TME is generally associated with a poorer 5-year event free survival in patients. Surprisingly, the presence of M2 macrophages in OS counteracts metastasis formation and increases the survival rate of high-grade OS patients [7, 399].

In addition, OS cells can produce EVs containing TGF-beta that activate local mesenchymal stem cells, which in turn release EVs containing IL-6, facilitating tumor progression. Furthermore, cytokine-containing EVs prepare the lung metastatic niche to receive OS circulating tumor cells and, acting as the main messenger between OS cells and the pulmonary parenchyma, contributing to the local tumor development [3]. Exosomes, nano-sized extracellular vesicles up to 100 nm in diameter, have a relevant cargo of miRNAs involved in cells crosstalk for physiological homeostasis maintenance, but also contributing to progression of different cancer types [400]. By releasing exosomes, tumor cells can reprogram their surroundings and shaping the TME into a tumor-permissive or tumor-promoting environment [401]. As an example specifically for OS, OS-derived exosomal miR-21 regulates the tumor microenvironment by targeting specific molecules in tumor cells, endothelial cells, cancer-associated fibroblasts and immune cells [17]. Indeed, exosomal miRNAs, secreted in different body fluids, on one hand represent a gold mine for identifying new diagnostic biomarkers, on the other hand represent a therapeutic opportunity by engineering them to deliver beneficial molecules [402]. In fact, cell–cell communication mediated by extracellular RNA is becoming increasingly appreciated, so much so that a data repository, the exRNA Atlas, has been created by the NIH Extracellular RNA Communication Consortium (https://exrna-atlas.org/), representing a resource for translational studies for diagnostics and therapeutics [403].

In particular, normalizing the TME may have therapeutic relevance, however, the high genetic heterogeneity of OS makes the TME much more complex than that of other tumors, and thus, potential TME normalizing drugs should have multiple targets. In this regard, exploring the RNA networks may pave the way for innovative therapeutic strategies. In fact, different studies highlighted the role of miRNAs in the crosstalk between OS cells and the TME; taking into consideration that the miRNA binding sites can be envisioned as the letters of an “RNA code”, the knowledge of ceRNETs involved in the communication between OS cells and the surrounding TME may offer the opportunity to manipulate them for normalizing TME. The combination of TME-normalizing drugs, including those RNA-based, and chemotherapy may offer promise for innovative therapeutic approaches.

Recent evidence revealed that also lncRNAs can be abundant and stable in EVs [404]. The lncRNA CASC15 was significantly upregulated in OS plasma exosomes as well as in OS tissues and cell lines (Table 1). Interestingly, CASC15 knockdown can restrain the proliferation, migration, and invasion of OS cells, and inhibit the growth of OS in xenograft models. Mechanistically, CASC15 is able to sponge miR-338-3p, thus up-regulating its oncogenic target RAB14; rescue experiments verified that CASC15 can promote OS cell growth and metastasis by upregulating RAB14 expression [43].

The role of macrophages-derived exosomal lncRNAs in osteosarcoma development has been studied in vitro by differentiating the human mononuclear cells THP-1 in tumor associated macrophages (TAMs) and then performing a high-throughput microarray assay to analyze the dysregulated lncRNAs and miRNAs in osteosarcoma cells co-cultured with macrophages-derived exosomes. Then, functional analyses revealed that macrophages-derived exosomal lncRNA LIFR-AS1 can be delivered to OS cells, and there its increased expression unbalance the ceRNET LIFR-AS1/miR-29a/NFIA, with the consequent promotion of cell proliferation, migration, invasion, and apoptosis inhibition [88] (Table 1). TAMs were also obtained by inducing CD14 + peripheral blood mononuclear cells (PBMCs); then, it was demonstrated that TAMs increased the lncRNA PURPL expression in OS cells, promoting cell proliferation, migration, invasion by miR-363/PDZD2 axis; this same axis can also modulate TAM migration, highlighting a possible feedback crosstalk between TAMs and OS cells [155] (Table 1).

As a critical component of TME, bone marrow-derived mesenchymal stem cells (BMSCs) have been demonstrated to modulate the cancer hallmarks. Li et al. demonstrated that BMSC-EVs facilitated proliferation, invasion and migration of osteosarcoma cells and promoted tumor growth in nude mice. In particular, BMSC can load MALAT-1 in EVs and deliver it to OS cells, potentially unbalancing all the ceRNETs mediated by the lncRNA. In particular, it has been demonstrated that BMSC-EVs-treated osteosarcoma cells showed increased MALAT1 and NRSN2 expressions, and activated Wnt/β-catenin pathway due to MALAT-1 sponging activity versus miR-143 [131]. BMSCs are also able to deliver the lncRNA NORAD through EVs to OS cells; there, NORAD promoted OS cell proliferation and invasion by sponging miR-30c-5p and thus increasing KLF10. Very importantly, these results were confirmed in vivo, where BMSC-EV-NORAD was also able to promote lung metastasis of osteosarcoma [145]. Intriguingly, KLF10 was also involved in another ceRNET, Circ-0003998/miR-197-3p/KLF10, promoting OS cell proliferative and invasiveness [230].

Finally, cytokines produced by different cell types in TME can increase the expression of oncogenic ncRNAs, derailing their governed network. One example is represented by cancer-associated fibroblasts (CAFs)-derived TGF-beta, that is able to upregulate the expression of TUG1 in OS cells [188]. There, up-regulated TUG1 is able to sponge different miRNAs, thus increasing the expression of their oncogenic targets prompting cell proliferation, migration, angiogenesis, tumor growth, and metastasis (Table 1).

**Fig. 2:**
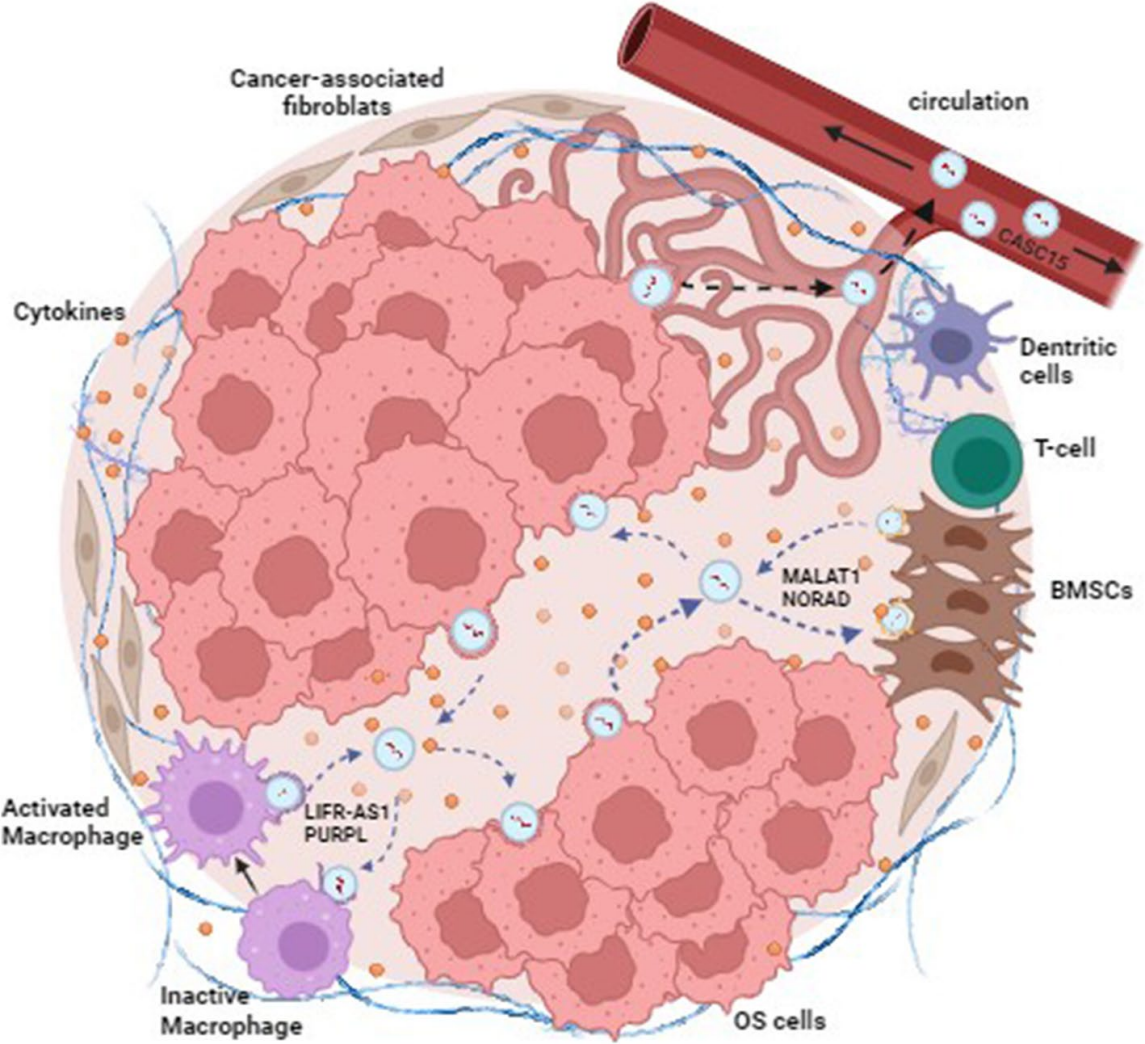
ceRNETs contributing to tumor microenvironment. A continuous crosstalk occurs among osteosarcoma cells (OS cells), bone mesenchymal cells (BMCs), immune cells, especially tumor-associated macrophages (TAFs), cancer-associated fibroblasts (CAFs) through cytokines and vesicles, with their cargo of proteins and RNAs, that can unbalance the RNA regulatory networks in the recipient cells, thus contributing to OS progression. Examples discussed in the text are reported. Figure created with BioRender.com

Overall, molecular mechanisms underlying the crosstalk among OS cells each other and the other components of TME are much more complex than expected and relying also on RNA regulatory networks that can be unbalanced in each cell type component by the surroundings (Fig. 2). These features are challenging for the comprehension of OS progression and patients’ management, but can offer innovative therapeutic opportunities, also in the direction of precision oncology.

## Conclusion and next challenges

OS is a very complex cancer: mesenchymal bone-forming cells can undergo aberrant alterations at any stage of differentiation; vast genomic instability and multiple genomic aberrations characterize the majority of OS cases; different mutated genes have been identified. The heterogeneity of genetic drivers and of cell types contribution to OS onset and progression, especially in TME, makes therapy and patients management particularly challenging. The deep understanding of OS biology and a unifying picture of molecular mechanisms could help to transform those challenges into opportunities. The ceRNET perspective may be a key to understand how different transcripts, coding and non-coding, are functionally linked and talk each other using the microRNA binding sites as the letters of an “RNA language”; indeed, the unbalancing of the networks can drive OS onset, progression and even chemotherapeutic resistance.

The knowledge of those mechanisms could inspire innovative therapeutic approaches based on restoring the optimal ceRNA crosstalk for the homeostasis equilibrium, with a view to achieving drugs for multiple targets, required by OS heterogeneity. It is becoming increasingly clear that RNA molecules as therapeutic agents are more cost-effective and easier to develop than traditional therapeutics based on small molecule chemicals or proteins, due to their structural/functional versatility allowing them to interact with DNA, other RNA biotypes and proteins and thus broadening the range of druggable targets. Different FDA and EMA drugs approved in clinical care or in clinical development cover the five different categories of RNA therapeutics, i.e.: mRNAs, RNA encoding for proteins; antisense oligonucleotides (ASOs), small single-stranded nucleic acids binding target RNA with perfect complementarity and thus inducing post-transcriptional gene silencing; small interfering RNAs (siRNAs), double-stranded RNA causing degradation or translational block of target RNAs; miRNA mimic or inhibitor, respectively small double-stranded RNA molecules boosting the miRNA level or small single-stranded RNA binding and suppressing the miRNA silencing activity; aptamers, RNA, DNA, or RNA/DNA hybrids that form secondary or tertiary structures binding to a target molecule, either suppressing or enhancing the pathway relying on that target [405, 406]. Even more RNA-targeted and RNA-based strategies have been found to have possible therapeutic potential, such as circRNA molecules carrying multiple binding sites for sponging oncomiRs and thus preventing their activity [407]. The basic concept is to restore the expression of beneficial molecules (such as a tumor suppressive miRNA) or silence the oncogenic molecules. Intriguingly, among the RNA therapeutics developed for other diseases, some strategies useful for OS can be found. One example is represented by MRX34, a miRNA mimic for miR-34a, that gained the Phase I of clinical trial for melanoma and other cancer types (NCT01829971), but that could be useful also for OS, due to its oncosuppressive activity [408]. Although the FDA halted the clinical trial for immune-related adverse events, it could be worth to develop other miR-34a mimic-based strategies, since its effectiveness has been also demonstrated by another miR-34a prodrug (chimeric recombinant tRNA fusion pre-miR-34a) that has anti-tumor activity just for OS, in a canine model [409]. Vice versa, RG-012 is a miR-21 inhibitor developed for Alport Syndrome (NCT03373786, Phase II), but that could be useful also for OS due to its oncogenic activity [410]. Other interesting approaches targeting lncRNAs, even for unrelated diseases and that could be inspiring and beneficial also for OS, have been found for the oncogenic lncRNA MALAT1 and the tumor suppressor lncRNA GAS5 (Table 1); in the first case, multiple structural element lockers are being developed for disrupting a stabilizing triple helix structure at its 3’ end, resulting in MALAT1 destabilization and downregulation; in the second case, the interaction element blocker, NP-C86 molecule, blocks the interaction with UPF1, which normally results in nonsense-mediated decay, thus increasing the stability and half-life of GAS5 [406, 411]. The next overcoming challenges for successful RNA therapy are probably represented by stable and possibly specific delivery of the molecule through the extracellular and intracellular barriers; for those obstacles, various chemical modifications and the engineering of delivery formulations have been explored to improve pharmacodynamics and pharmacokinetics. In particular, five nanocarriers delivery strategies have been developed, i.e. lipid nanoparticles, cationic polymers, engineered exosomes, spherical nucleic acid nanoparticles, self-assembled DNA cage tetrahedron nanostructures, and they can keep the promise to deliver RNA molecules through binding to the cell membrane, endocytosis, endosome escape and release RNAs in the cytoplasm for translation or incorporation into appropriate ribonucleoproteins complexes [407].

Indeed, the combination of RNA-targeted and RNA-based therapies with lower doses of current treatments could be exploited, also in an attempt to normalize TME, inhibit metastasis, prevent or overcome the chemoresistance, in the perspective of personalized therapeutic plans.

Finally, many RNAs discussed here and listed in the Tables are consistently reported as up-regulated or down-regulated in OS tissues and cells, and related to tumor stage, progression, prognosis and survival. It would be worth setting-up PCR arrays for simultaneously and systematically measuring, in large cohorts, the different candidates to find those ones useful as biomarkers for an early diagnosis, prognosis and monitoring therapy response. Different diagnostic panels are now commercially available for various diseases, including cancer, and more than 150 clinical studies are registered at clinicaltrials.gov, wherein the value of a miRNA or miRNA signature is being investigated for a variety of clinical applications from early disease detection to treatment response [412–414]. A clear trend in the recent literature and on-going clinical trials can be envisaged, that is the development of miRNA-based noninvasive detection assays using liquid biopsies (mainly blood or serum samples) as starting material to inform clinical decisions, whereas initial diagnostic/prognostic studies used tissues from diagnostic biopsies or surgical procedures. Some challenges with circulating RNA-based diagnostic applications are related to their specificity, since certain miRNAs can be altered in other physiological (e.g., pregnancy) and pathological conditions and their diagnostic performance could be lower compared to other investigational and clinically established biomarkers; however, combining the detection of different RNA molecules, for example involved in various ceRNETs consistently reported to be related to tumor stage, progression, prognosis, survival and therapy response, may be the linchpin to overcome the problems. Those approaches will be useful in the near future for defining new diagnostic tools and supporting precision oncology.

Overall, a multidisciplinary approach, based on deep knowledge crossing the field of both RNA and cancer biology, is increasingly required, especially when the subject of study is so complex and heterogenous such as OS and patients care.

## Data Availability

Not applicable.
